# Non‐Specific Particle Formation During Extracellular Vesicle Labelling With the Lipophilic Membrane Dye PKH26

**DOI:** 10.1002/jev2.70079

**Published:** 2025-05-19

**Authors:** Laurel A. Haines, Alex A. Baeckler, Sophi J. Schofield, Eric P. Palmer, Bradley F. Guilliams, Melinda A. Meyers, Daniel P. Regan

**Affiliations:** ^1^ Department of Microbiology, Immunology, & Pathology College of Veterinary and Biomedical Sciences Colorado State University Fort Collins Colorado USA; ^2^ Department of Clinical Sciences College of Veterinary Medicine North Carolina State University Raleigh North Carolina USA; ^3^ Department of Chemistry College of Natural Sciences Colorado State University Fort Collins Colorado USA; ^4^ Analytical Resources Core Center for Imaging and Surface Science Colorado State University Fort Collins Colorado USA; ^5^ Department of Clinical Sciences College of Veterinary and Biomedical Sciences, Colorado State University Fort Collins Colorado USA

**Keywords:** extracellular vesicles, labelling, lipophilic membrane dye, PKH26, tracking

## Abstract

Current approaches for the fluorescent labelling of extracellular vesicles (EVs) have been reported to produce widely variable and controversial results, highlighting a significant need for validated, reproducible labelling methods to advance the field of EV research. Lipophilic membrane dyes are commonly used but have been shown to produce non‐specific fluorescent particles that are indistinguishable from labelled EVs, confounding experimental results. We aimed to distinguish conditions that can either promote or reduce the formation of non‐specific dye particles when using the prototypical lipophilic membrane dye PKH26. We optimised a labelling approach that minimises the production of non‐specific dye particles by altering buffer conditions during staining and validated this method across cell‐based and in vivo systems of EV biodistribution. To do this, we specifically isolated small EVs using ultrafiltration and size exclusion chromatography and validated sample purity and post‐isolation processing steps. We then used single‐EV spectral flow cytometry and transmission electron microscopy to investigate the impact of four different buffer conditions on PKH26 non‐specific particle formation. We also determined the extent to which non‐specific PKH26 particles were detectable in cell‐based assays and in vivo within mouse lymph nodes using flow cytometry, immunofluorescence, and intravital imaging. By optimising buffer conditions to eliminate additional protein, we were able to minimise the formation of dye aggregates while maintaining efficient EV labelling, producing a much higher signal‐to‐noise ratio both in vitro and in vivo. We also demonstrate that failure to include proper vehicle controls can have significant implications on experimental results, leading to false positive data. This work emphasizes the importance of adequately benchmarking EV labelling approaches as it is essential for accurate evaluation of EV trafficking in physiologic and pathologic states.

## Graphical abstract

Lipophilic membrane dyes, like PKH26, have been associated with the production of non‐specific fluorescent dye particles that can confound experimental results. We aimed to investigate conditions that can minimise these non‐specific signals while maintaining efficient labelling of true extracellular vesicles. We identified that the addition of protein during the staining process can lead to substantial formation of dye‐protein aggregates that cannot be removed using traditional approaches. We validated an optimised PKH26 staining approach across both in vitro and in vivo systems.

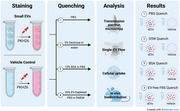

## 1 Introduction

Extracellular vesicle (EV) research has been rapidly expanding across a range of scientific disciplines, particularly in the field of cancer biology. There has been a nearly 30‐fold increase in the number of publications containing the terms ‘extracellular vesicle’ and ‘cancer’ in just the last decade (National Center for Biotechnology Information 1988). Investigations into the biodistribution of EVs during cancer progression and the trafficking of nanoparticle drug therapies are of growing interest (Kim et al. 2021; Kong et al. 2021; Costa‐Silva et al. 2015; Tian et al. 2014; Bellavia et al. 2017). Among published literature on EV trafficking and cellular uptake, there remains considerable heterogeneity in the methods used for EV labelling. It has been increasingly noted that even minor variations in labelling protocols can dramatically alter experimental outcomes (Simonsen 2019; Théry et al. 2018; Welsh et al. 2024; Takov et al. 2017). These findings emphasise the importance of understanding the caveats of a given EV labelling protocol and employing appropriate controls and validation steps prior to experimental interpretation.

The 2023 update to the *Minimal information for studies of extracellular vesicles* (MISEV2023) provides a comprehensive resource for EV investigators and has helped standardise the field of EV research (Welsh et al. 2024). MISEV2023 contains specific suggestions as to the necessary information needed to adequately isolate and verify EV preparations. Moreover, it expands on the 2018 MISEV guidelines to include details about specific EV labelling approaches and some of the drawbacks associated with different methodologies (Théry et al. 2018; Welsh et al. 2024). The guidelines do not put forth a recommendation for the most appropriate EV labelling approach, but they do identify critical controls and suggest that a multi‐pronged labelling approach may improve specificity (Welsh et al. 2024). Both the 2018 and 2023 updates to the MISEV guidelines emphasise the importance of dye vehicle controls and thorough validation of any EV labelling approach (Théry et al. 2018; Welsh et al. 2024).

Of the EV labelling methodologies, there have been growing concerns over the use of lipophilic membrane dyes (LMDs), also known as lipid‐anchored fluorophores, to label EVs (Simonsen 2019; Théry et al. 2018; Takov et al. 2017; Melling et al. 2022; Chen et al. 2023; Dehghani et al. 2020; Pužar Dominkuš 2018). However, LMDs continue to be widely used in EV research with substantial variation in labelling protocols and validation controls (Costa‐Silva et al. 2015; Ruan et al. 2024; Guo et al. 2022; Wei et al. 2020; Herman et al. 2023). Thus, it is critically important that these dyes be thoroughly evaluated and specific recommendations made regarding their appropriate use and necessary controls. Though the concerns with LMDs have been proposed in the literature, there remains limited published reports of the true implications that different LMD protocols have on in vitro and in vivo experimental systems.

One of the major concerns surrounding the use of LMDs is their propensity to form non‐specific dye particles in aqueous solutions due to their hydrophobic nature (Simonsen 2019; Takov et al. 2017; Melling et al. 2022; Pužar Dominkuš 2018). Dye particles can result from the self‐aggregation of LMDs into EV‐like nanoparticles or micelles (Pužar Dominkuš 2018). However, they can also react with contaminating proteins and lipoproteins in EV preparations, forming non‐vesicular, fluorescent particles that are not surrounded by a lipid bilayer like EVs (Simonsen 2019; Takov et al. 2017). Plasma proteins like albumin or very low‐density lipoprotein (VLDL) can commonly contaminate EV preparations and have hydrophobic binding sites that can bind lipids and thus, may bind LMDs as well (Simonsen 2019; Chen et al. 2023; Karimi et al. 2018; Curry et al. 1998). Although prototypical LMDs are intended to become stably incorporated into membranes, it has been suggested that desorption of dyes from these membranes and subsequent binding by surrounding proteins is possible (Simonsen 2019). In fact, many protocols, including manufacturer suggestions, include the application of ‘stop solution’ to labelled cells to quench unbound dye such as 10% bovine serum albumin (BSA) or foetal bovine serum (FBS) (Chen et al. 2023; Pužar Dominkuš 2018; Tario et al. 2011). When staining cells, the LMD‐protein particles formed during this step can be isolated away from cells with centrifugation steps; however, these particles can appear similar in size to EVs, so when labelling EVs with LMDs, it poses a major challenge to differentiate labelled EVs from LMD‐protein aggregates (Simonsen 2019; Tario et al. 2011).

Non‐specific LMD particles can be internalised by cells and appear indistinguishable from labelled EVs (Simonsen 2019; Melling et al. 2022; Pužar Dominkuš 2018). Sucrose gradients or size exclusion chromatography have been proposed as approaches to remove LMD particles; however, they can result in low sample recovery and introduce more variability than traditional approaches that rely on ultracentrifugation (Pužar Dominkuš 2018). Moreover, these non‐specific LMD particles can lead to erroneous positive results if appropriate dye vehicle controls are not included. It is essential to investigate techniques that minimise the production of these particles and understand proper controls to correct for the level of non‐specific LMD particle contamination when reporting results.

Here, we evaluate the impact of four different labelling protocols on the formation of non‐specific LMD particles by the widely used PKH family of LMD dyes. We investigate the impact of each labelling approach and the associated non‐specific particles in both cell‐based and in vivo experimental systems. To achieve these experimental aims, we isolated small EVs (sEVs) (<200 nm average diameter) from osteosarcoma tumour cell lines using a two‐step approach of ultrafiltration followed by size exclusion chromatography. We then evaluated sEV labelling using PKH26 via nanoparticle tracking analysis, spectral flow cytometry and intravital imaging.

This work provides an experimental blueprint for the evaluation and validation of different EV labelling approaches. Importantly, our work shows how specific experimental steps result in the considerable production of non‐specific LMD particles that are indistinguishable from labelled EVs. By optimising staining conditions, we can minimise the formation of PKH26 dye particles in control samples while maintaining robust EV labelling, allowing for reproducible evaluation of in vitro and in vivo EV uptake. These findings emphasise the need to fully characterise and report EV labelling approaches in order to understand potential pitfalls. Doing so is critical for the downstream evaluation of EV trafficking in the context of cancer biology and across scientific disciplines.

## 2 Methods

### 2.1 Cell Culture

All cell lines were obtained from the American Type Culture Collection (ATCC) and confirmed to be negative for mycoplasma every month using a MycoAlert detection kit (Lonza). Cell lines include the human osteosarcoma line 143B, the murine osteosarcoma line K7M2, and the human monocytic cell line THP‐1. Cells were maintained in complete Dulbecco's Modified Eagle Medium (DMEM) supplemented with 10% foetal bovine serum and Penicillin‐Streptomycin (10 U/mL) with the exception of THP‐1, which was maintained in RPMI 1640 with 10% FBS and penicillin‐streptomycin (10 U/mL). All cells were used for EV collection or experiments within 10 passages of their initial start date.

### 2.2 sEV Isolation

**FIGURE 1:**
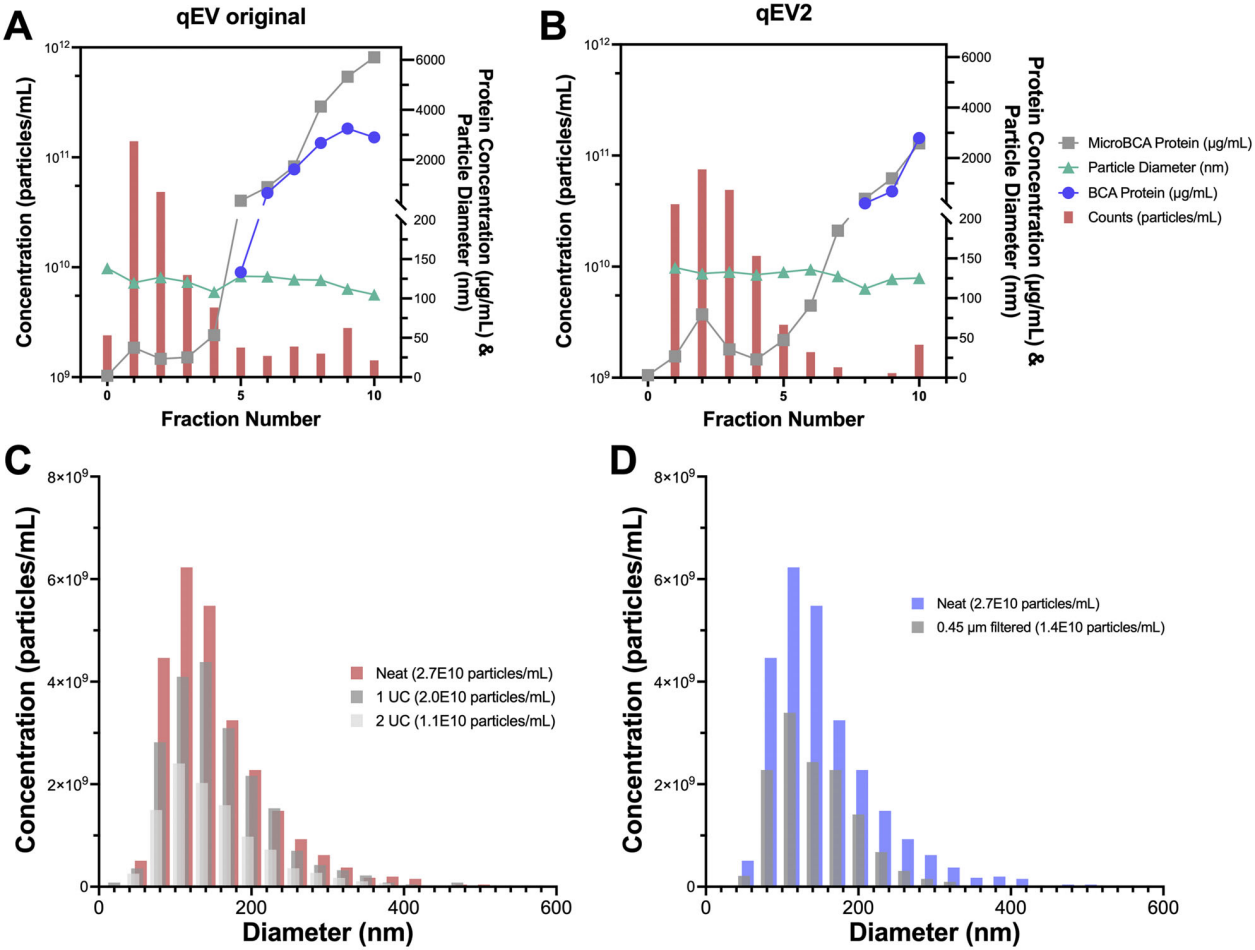
Characterisation of sEV isolation approach and the impact of post‐isolation processing steps. sEVs were isolated from ∼200 mL of tumour cell (143B) conditioned media and processed via ultrafiltration using 100 kDa cut‐off filters followed by size exclusion chromatography using the Izon Automated Fraction Collector. Isolated fractions were assessed from both the qEV original column (A) and the qEV2 column (B). Particle counts per millilitre (pink columns), particle diameter in nanometre (green triangles), and protein concentration (µg/mL) determined via BCA (blue circles) and microBCA (grey squares) were assessed across all fractions and the column void volume (labelled as fraction 0) to determine purity and quantity of collected particles (A, B). (C) The impact of two ultracentrifugation steps, as used during sEV labelling approaches, was determined by assessing sEV concentration and diameter via nanoparticle tracking analysis. Fractions 1 through 4 from the qEV2 column were pooled to obtain 143B‐derived sEVs for this analysis. The original sEV isolate (pink columns, ‘Neat’) is compared to the same isolate receiving one ultracentrifugation at 100,000 × *g* for 70 min (dark grey columns, ‘1 UC’), or two centrifugation steps (light grey columns, ‘2 UC’). (D) Similarly, 143B sEVs were prepared by pooling the first four fractions from the qEV2 column and measured via nanoparticle tracking analysis (blue columns, ‘Neat’), and then the sEV suspension was filtered through a pre‐wetted, 0.45 µm PES syringe filter to show the reduction in particle counts (grey columns, ‘0.45 µm filtered’).

sEVs were isolated from 143B (human) and K7M2 (mouse) cell lines using a two‐step isolation approach of ultrafiltration and size‐exclusion chromatography. Cells were grown in T‐225 flasks and were passaged at least once prior to sEV collection. After reaching 50%–70% confluence, cells were washed once with sterile PBS (without calcium and magnesium, Corning) and the media replaced with serum‐free DMEM. Media was collected when cells reached 90%–100% confluence within 48 h after media replacement. Media was spun at 300 × *g* for 5 min to remove contaminating cells and frozen at –20°C for future EV isolation (supernatants used within 2 months after freezing). To isolate sEVs from conditioned media, media was thawed and ∼200 mL was pooled together following an additional spin at 2000 × *g* for 10 min to remove debris. Conditioned media was then subjected to ultrafiltration using Amicon Ultra Centrifugal filters (Centricon Plus‐70) with a 100 kDa molecular weight cut‐off to concentrate EVs (3500 × *g*, 40 min per 70 mL). These particle concentrates were then washed once with an additional 25 mL of sterile PBS in the same Centricon filter (3500 × *g*, 15 min). To isolate sEVs from these concentrated particles, the concentrate was separated by size‐exclusion chromatography (SEC) using the qEV automated fraction collector (Izon Science). All 10 fractions as well as the void volume were evaluated for two different SEC column types in a PBS buffer: the qEVoriginal (Generation 2, 35 nm, 0.5 mL sample volume, 0.4 mL fraction size) and qEV2 (Generation 2, 35 nm, 2 mL sample volume, 2 mL fraction size) in Figure 1. For all other experiments, the first four fractions from the qEV2 column were pooled for downstream use. Columns were reused no more than five times per manufacturer recommendation and washed with PBS three times between uses. For all sEV‐free controls, PBS alone was run through the qEV2, and the first four fractions were pooled to match the processing steps for sEVs. All sEVs were used fresh without freezing following elution from the column, stored in PBS at 4°C, and discarded after 1–2 weeks to limit EV deterioration (Su et al. 2021; Ahmadian et al. 2024).

### 2.3 sEV Particle and Protein Quantification

EVs were quantified by nanoparticle tracking analysis (NTA) using a ZetaView QUATT 4 NTA from Particle Metrix GmbH. Briefly, EV preparations were diluted in 0.22 µm filtered distilled water at room temperature, for an average count of 50–500 particles per frame. The diameter and quantity of EVs were determined using Brownian motion. Measurements to determine the size distribution and concentration were taken at 11 positions via scatter mode using a 488 nm laser. Raw and summary data were exported to analyse particle diameter and concentration using the Zetaview Software (version 8.05.12 SP1) and GraphPad Prism (version 10.2.2). Total protein for each SEC fraction, or pooled sEV preparations, was analysed without lysis of EVs to determine protein contamination levels via bicinchoninic acid (BCA) assay and/or microBCA (Pierce) according to manufacturer instructions (Smith et al. 1985). To evaluate the impact of different post‐processing steps, sEVs were ultracentrifuged for 70 min at 100,000 × *g* at 4°C on a benchtop Optima MAX‐XP Ultracentrifuge (Beckman Coulter). sEV preparations were also subjected to syringe filtration to evaluate the impact on sample loss through a 13 mm, 0.45 µm PES filter (Foxx Life Sciences) that had been pre‐wetted with sterile PBS.

### 2.4 sEV Characterisation via Transmission Electron Microscopy (TEM)

sEVs were imaged within 2 days of isolation and the same day as staining with PKH26 (described in Section 2.7) for stained samples and dye vehicle controls. 5 µL of a 5E10/mL sEV suspension was pipetted onto a 5–6 nm thick carbon‐coated 200‐mesh copper grid (Electron Microscopy Sciences) and allowed to incubate for 3 min and excess liquid was blotted off. This process was repeated for a total of 10 µL of sEVs amounting to 5E8 total particles loaded. Adhered EVs were washed twice with ultra‐pure water for 1 min each to minimise salt contamination, and excess liquid was removed. Next, the adhered sEVs were negatively stained with 5 µL of 2% uranyl acetate for 2 min (1% final concentration), blotting and applying stain again after 5, 15, 30, and 60 s. At 2 min, the adhered sEVs were washed twice for 1 min each with ultra‐pure water to remove excess stain. Excess ultra‐pure water was blotted and left to air‐dry for 5 min. sEV unstained samples and PKH26‐stained sEVs were imaged neat, and dye control samples, or a matched unstained BSA sample, were diluted 10‐fold prior to imaging due to high amounts of albumin contamination. sEV preparations were imaged from several different grid squares at random on a JEOL JEM‐2100F Transmission Electron Microscope housed at the Analytical Resources Core at Colorado State University.

### 2.5 sEV Characterisation via Western Blot

To evaluate sEV surface marker expression and purity, we compared an equivalent volume of 143B sEVs pooled from fractions 1 through 4 from the SEC column to several additional control samples: fractions 5 through 10, a protein solution that had passed through an ultrafilter (100 kDa cut‐off), or a 143B whole cell lysate. Samples were sonicated three times for 5 min at room temperature using a Elmasonic Easy 20 H ultrasonic bath (37 kHz ultrasonic frequency). Samples were combined with 4X Laemmli buffer (Bio‐Rad) containing 5% 2‐mercaptoethanol for a final concentration of 1X Laemmli buffer and boiled at 95°C for 5 min. Samples were then loaded onto a Mini‐PROTEAN TGX Precast Protein Gel (10% polyacrylamide, Bio‐Rad) and run with a molecular weight ladder (Precision Plus Protein Dual Colour Standard, Bio‐Rad) for ∼1.5 h at 150 V. The protein gel was transferred to a PVDF membrane (Bio‐Rad) that had been washed in methanol for 30 s using a Trans‐Blot Turbo transfer system (Bio‐Rad), blocked for 1 h at room temperature using 5% BSA in tris‐buffered saline (TBST) (20 mM Tris base, 150 mM NaCl, 0.1% Tween20), and then incubated overnight at 4°C with a primary antibody in 5% BSA in TBST. Primary antibodies used include: anti‐CD9 clone D8O1A rabbit monoclonal, 1:2000 dilution (Cell Signalling Technology, Catalog Number 13174), anti‐CD81 clone D3N2D rabbit monoclonal, 1:1000 dilution (Cell Signalling Technology, Catalog Number 56039) and anti‐GM130 clone D6B1 rabbit monoclonal negative control antibody at 1:5000 (Cell Signalling Technology, Catalog Number 12480). An anti‐rabbit secondary antibody conjugated to horseradish peroxidase (Cell Signalling Technology, Catalog Number 7074) was then added after three wash steps with TBST for 1 h at room temperature and then, after another three washes in TBST, Super Signal West Dura Extended Duration Substrate (ThermoFisher Scientific) was added per manufacturer instructions prior to imaging the membrane with a ChemiDoc XRS+ Imaging System (Bio‐Rad). Although samples were matched based on volume, protein concentration was determined via micro‐Bicinchoninic acid assay (Pierce) to ensure gel was not overloaded, the final protein present in each of the volume matched samples loaded onto the gel was: 1.52 µg (fractions 1–4), 3.75 µg (fractions 5–10), 10 µg (flow‐through), and 2 µg (143B cell lysate) (Smith et al. 1985).

### 2.6 sEV Characterisation via Single‐EV Flow Cytometry

To assess the surface marker expression of single EVs by flow cytometry, we analysed purified sEV samples, control SEC‐isolated PBS samples, and polystyrene sizing beads (Spherotech). sEV samples were isolated as described above (Section 2.2) and analysed for flow cytometry fresh, never frozen and maintained at 4°C unless otherwise specified. To evaluate canonical EV surface marker expression, 1E10 sEVs per sample were blocked with either human Fc‐Receptor block (eBioscience) or serum‐free mouse block (human IgG, Jackson ImmunoResearch Labs and rat anti‐mouse CD16/CD32, eBioscience) for 20 min on ice. Next, an equal volume of PBS containing the antibodies of interest was added to obtain a final dilution of 1:100 and incubated in the dark for 20 min at room temperature. Antibodies and isotypes include: Pacific Blue anti‐human CD63 (clone H5C6, BioLegend), mouse IgG1 κ Pacific Blue isotype control (clone MOPC‐21, BioLegend), FITC anti‐human CD81 (clone M38, Invitrogen), mouse IgG1 FITC isotype control (VI‐AP, Invitrogen), FITC anti‐mouse CD63 (NVG‐2, BioLegend), rat IgG2a κ FITC isotype control (clone RTK2758, BioLegend) and APC anti‐mouse CD81 (clone Eat‐2, BioLegend), or Armenian Hamster IgG1, κ APC isotype control (clone HTK888, BioLegend). Samples were then washed with 1 mL of sterile PBS by ultracentrifuging at 100,000 × *g* for 70 min at 4°C. Samples were resuspended in 150 µL of PBS prior to reading on the cytometer. Unstained controls for each EV type, a buffer (PBS) only control, single stained controls, fluorescence‐minus‐one controls (where applicable) and isotype controls were included for each sample. Samples were analysed on an Aurora 4‐laser Spectral Cytometer (Cytek Biosciences) with threshold settings lowered and gain settings increased to detect nanosized particles. The cytometer passed a daily quality control test using Cytek QC Beads to determine appropriate fluorescent laser parameters and received monthly preventative maintenance. Gates were set based on particle side scatter parameters to include particles between ∼113 and ∼450 nM as determined using the polystyrene sizing beads. To assess particle counts between samples for sEVs labelled with PKH26, the cytometer was set to collect an equal volume of 10 µL per sample, and the cytometer completed two rinse cycles between each sample to limit cross contamination between sEVs and dye controls. To evaluate total particle counts per mL, the gating strategy included all particles, just those between 113 and 450 nM. To determine the percent of PKH26 sEVs that were also positive for CD63 and CD81 surface markers, we first labelled sEVs with these antibodies or matched isotype controls and then stained sEVs with PKH26 as described in Section 2.7.

### 2.7 sEV Labelling With PKH26

Based on the starting sEV concentration, 1E10 total sEVs were centrifuged at 100,000 × *g* for 70 min at 4°C on a Beckman Coulter MAX‐XP Benchtop Ultracentrifuge. As a matched control for PKH26 labelling, sEV‐free, sterile PBS was prepared identically as the sEVs samples, including a processing step through the SEC column and then concentrating the same volume of the sEV‐free PBS control on the ultracentrifuge. Following ultracentrifugation, supernatants were removed, retaining 20 µL of sEVs or control samples, and the pellets were resuspended by pipetting (15 times), vortexing for 30 s, and then placing on an orbital shaker at 1500 rpm for 5 min at room temperature. Samples were then resuspended in an additional 80 µL of diluent C (Sigma–Aldrich, Catalog Number: CGLDIL) by pipetting up and down 15 times at room temperature followed by 30 s of vortexing. Samples were then diluted with an equal volume (100 µL) of 2 µM PKH26 (Sigma–Aldrich, Catalog Number: P9691) in diluent C for a final staining concentration of 1 µM as this has been shown to reduce self‐aggregation of PKH26 into micelles while still having high labelling efficiency (Chen et al. 2023). Though lower than manufacturer's recommendation, we observed a strong PKH26 signal with 1 µM and chose to proceed with this concentration to minimise excess unincorporated PKH26 dye. After 5 min of staining time, samples were transferred directly into a new ultracentrifuge tube containing 1 mL of the specified quenching buffer. Samples were quenched with 1 mL of either sterile PBS, sterile 5% dextrose in water (D5W), 10% bovine serum albumin (BSA) in PBS or 20% EV‐depleted FBS (Gibco) in DMEM containing streptomycin and penicillin. All samples were ultracentrifuged again at 100,000 × *g* for 70 min at 4°C and resuspended in 200 µL sterile PBS for downstream use. For comparison of ultracentrifugation to a column‐based approach for dye particle removal from stained samples, stained sEV samples and dye controls were processed through Exosome Spin Columns (MW 3000, Invitrogen) and compared to ultracentrifugation alone.

### 2.8 In Vitro Assessment of sEV Uptake by Myeloid Cells

Human (THP‐1) monocytes were cultured in 96‐well plates for 24 h at a density of 75,000 cells per well and then treated with 1E9/mL PKH26‐labelled sEVs or a matched PKH26 dye control for either 4 or 24 h at 37°C in the dark in serum‐free RPMI supplemented with 1% penicillin/streptomycin. Cells were then washed twice with PBS and transferred into a 96‐well V‐bottom plate for preparation for flow cytometry analysis (described in Section 2.11). Samples were run in biological quadruplicate (four independent experimental days).

### 2.9 Fluorescent Microscopy of THP‐1 Cell sEV Uptake

THP‐1 monocytes were differentiated into a macrophage state using 20 ng/mL phorbol 12‐myristate 13‐acetate (PMA) for 24 h on glass coverslips to encourage adherence. After 24 h, cells were treated with 1E9/mL PKH26‐labelled sEVs or dye control samples for another 24 h in the presence of PMA. Coverslips were then washed twice with PBS, and samples were fixed for 10 min in 2% paraformaldehyde in PBS. Samples were then washed twice and incubated with 100 mM glycine in PBS for 10 min to reduce autofluorescence. After an additional two washes with PBS with 0.05% Tween20 (PBST), cells were incubated for 30 min with ActinGreen 488 Phalloidin (ThermoFisher). The cells were then incubated with sterile filtered 1 µg/mL DAPI (ThermoFisher) in PBS for 10 min followed by another two washes with PBST. Finally, coverslips were mounted onto glass slides using Diamond Antifade Mountant (ThermoFisher) and imaged for fluorescence on an Olympus Confocal Microscope housed at the Animal Cancer Center at Colorado State University.

### 2.10 In Vivo Detection of sEVs and PKH26 Dye Particles in a Murine Model

Six‐ to eight‐week‐old female CD‐1 mice were purchased from Charles River Laboratories. Mice were housed in microisolator cages in laboratory animal staff‐managed facilities at Colorado State University, and all animal procedures were approved by the Institutional Animal Care and Use Committee at Colorado State University. To assess how sEV labelling techniques impact in vivo sEV biodistribution and cellular uptake, we injected 2.5E10 PKH26‐labeled murine K7M2 sEVs, a concentration visible in the draining lymph nodes by 24 h post‐injection by intravital fluorescence imaging (IVIS Spectrum In Vivo Imaging System), or a matched PKH26 dye vehicle control, resuspended in 50 µL sterile PBS into the footpad of 6‐ to 8‐week‐old, female CD‐1 mice. Each mouse received PKH26‐labelled EVs in one foot pad and a matched dye control in the other footpad. After 24 h, we surgically dissected the popliteal lymph nodes and measured ex vivo fluorescence with the IVIS Imaging System (excitation peak 551 nm, emission peak 564 nm). Subsequently, the lymph nodes were placed in 10 mL of ice cold DMEM containing 10% FBS and mechanically separated into a single cell suspension using the rubber end of a 3 mL syringe to filter the cells through a 70 µm cell strainer. Cell suspensions were spun at 300 × *g* for 5 min at 4°C and then resuspended and spun again in another 10 mL of DMEM. Cell suspensions were resuspended in a final volume of 1 mL complete DMEM for counting using a Cellometer Automated Cell Counter (Nexcelom). 250,000 cells per sample were processed for flow cytometry as described in Section 2.11.

### 2.11 Flow Cytometry of Cell Suspensions

Single cell suspensions of THP‐1 or mouse popliteal lymph node cells were plated in a 96‐well V‐bottom plate. Cells were then resuspended in 100 µL of the live/dead dye Zombie NIR (1:1000 dilution in PBS) in the dark at room temperature for 15 min. Cells were then washed three times in sterile flow cytometry buffer (sterile PBS with 1% bovine serum albumin, 0.05% sodium azide and 5 mM EDTA). THP‐1 cells were then immediately analysed on a 4‐laser Aurora Spectral Cytometer 16V‐14B‐10YG‐8R (Cytek Biosciences). Isolated lymph node cells were blocked for 10 min at room temperature with 10 µL mouse Fc block (normal mouse serum (Jackson ImmunoResearch Labs, Catalog# 015‐000‐120), ChromPure Human IgG (Jackson ImmunoResearch, Catalog# 009‐000‐003) and rat anti‐mouse CD16/CD32 Monoclonal Antibody (eBioscience, Catalog# 16‐0161‐82)). After blocking, cells were incubated with a lymph node antibody panel for 20 min at room temperature in flow cytometry buffer. Anti‐mouse antibodies in the lymph node panel include: BV510‐anti‐B220/CD45R (clone RA3‐6B2, BioLegend, Catalog# 103247, dilution factor of 1:200), BV605‐anti‐CD169 (clone 3D6.112, BioLegend, Catalog# 142413, 1:200), BV785‐anti‐Ly6C (clone HK1.8, BioLegend, Catalog# 128041, 1:300), PerCP‐anti‐CD11b (clone M1/70, BioLegend, Catalog# 101223, 1:100), FITC‐anti‐CD45 (clone 30F11, BioLegend, Catalog# 101229, 1:400), PerCP‐eFluor710‐anti‐CD31 (clone 390, ThermoFisher, Catalog# 46‐0311‐80, 1:300), PE‐Cy7‐anti‐CD11c (clone N418, BioLegend, Catalog# 117317, 1:200) and APC‐anti‐CD3 (clone 145‐2C11, CytekTonbo, Catalog# 20‐0031‐U025, 1:200). Single‐stained controls of untreated mouse lymph nodes were used as references controls for spectral unmixing and fluorescence‐minus‐one controls were used to determine appropriate gating schemes. Following staining, lymph node cells were washed three times and resuspended in flow cytometry buffer prior to analysis on the cytometry. Data was unmixed using SpectroFlo software and fcs files were further analysed in FlowJo, version 10.10.0.

### 2.12 Statistical Analyses

All analyses were performed in GraphPad Prism, version 10.2.2. Comparisons between PKH26 quenching methods for single sEV flow cytometry were first assessed for normality a Shapiro‐Wilk test (*α* = 0.05), and then subsequently analysed using a ratio paired *t*‐test and corrected for multiple comparisons using the two‐stage step‐up method of Benjamini, Krieger and Yekutieli with a false discovery rate of <5% (Benjamini et al. 2006; Shapiro and Wilk 1965). To assess in vitro cell uptake, we evaluated differences in treatment groups following the Shapiro‐Wilk test for normality by using an unpaired *t*‐test with a Welch's correction with the two‐stage step‐up method of Benjamini, Krieger and Yekutieli with a false discovery rate of 5% for multiple comparisons (Benjamini et al. 2006). For the assessment of in vivo uptake data via IVIS imaging or flow cytometry, multiple unpaired *t*‐tests (*α* = 0.05) were performed.

## 3 Results

### 3.1 Characterisation of a Two‐Step sEV Isolation Approach and the Impacts of Post‐Isolation Ultracentrifugation and Sterile Filtration

We determined the impact of a two‐step isolation process on sEV concentration, diameter and protein content. sEVs were isolated from conditioned media from murine (K7M2) or human (143B) osteosarcoma cell lines after 48 h of growth in EV‐free media when cells reached 90%–100% confluence. Conditioned media was centrifuged to remove cell contamination and debris and then concentrated using a 100 kDa cut‐off Centricon Ultra‐70 ultrafiltration device. sEVs were isolated from concentrates via size exclusion chromatography (SEC) using the Izon Automated Fraction Collector (AFC). Two different SEC columns were compared: the qEV original (Figure 1A) and the qEV2 (Figure 1B). Particle quantity and size distribution were assessed for each fraction from the SEC columns using nanoparticle tracking analysis. The protein concentration of each fraction was determined via BCA and microBCA without lysis of sEVs.

The majority of sEVs eluted in the first four fractions, with little to no sEVs eluting in the void volume (fraction 0). The protein concentration rises in later fractions but remains low across fractions 1 through 4 where particle counts are highest (Figure 1A,B). It has been noted by others that relatively pure populations of sEVs have low contaminating protein levels, which supported our decision to pool fractions 1 through 4 as our sEV isolate (Webber and Clayton 2013). The average diameter of sEVs fractions 1 through 4 was 119.05 nm for the qEV original and 133.05 nm for the qEV2, both within the size range for categorisation as sEVs (<200 nm) (Théry et al. 2018). Because of the high particle concentration, average diameter of <200 nm, and low protein contamination, we chose to pool fractions 1 through 4 for use in subsequent analyses of sEVs and proceeded with the qEV2 due to its higher volume yield. We evaluated sEV fractions isolated from the qEV2 both in human 143B cell line derived sEVs (Figure 1B) as well as murine K7M2 cell line derived sEVs (Figure S1A) to ensure reproducibility across cell lines.

Next, we investigated the impact of commonly employed post‐isolation processing techniques on sEV preparations. First, to mimic the steps taken during sEV fluorescent labelling with PKH26 and commonly employed in EV processing, we subjected sEVs to either one or two ultracentrifugation steps at 100,000 × *g* for 70 min (Livshits et al. 2015). We observed that on average, ultracentrifugation resulted in a ∼1.6‐fold decrease in sEV counts but did not dramatically impact sEV diameter, with only a minimal increase in mean diameter from 148.0 to 150.4 nm (Figure 1C). Moreover, we investigated the impact of including a filtration step using a 0.45 µm syringe filter with a polyethersulfone (PES) hydrophilic membrane on sEV concentration and size, as certain experiments may necessitate the need for sterile conditions. We observed a 1.75‐fold decrease in sEV concentration with 0.45 µm filtration with a minor impact on EV diameter, with an observed decrease from 148 to 138.7 nm (Figure 1D). For sEVs used in subsequent studies, we avoided filtration through a 0.45 µm filter to ensure we had appropriate sEV yield. However, we chose to proceed with the use of ultracentrifugation during staining with PKH26 as we deemed this a necessary step in the washing process and it had minimal impact on sEV diameter.

### 3.2 Confirmation of Canonical sEV Marker Expression and sEV Morphology

**FIGURE 2:**
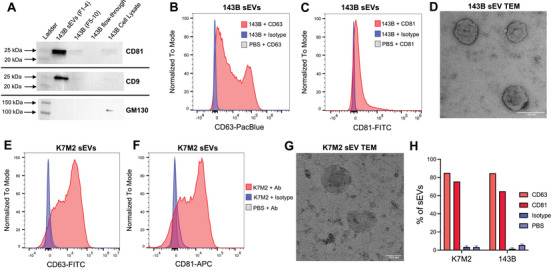
Determination of surface marker expression and morphology using Western blot, single‐EV flow cytometry and transmission electron microscopy. (A) Isolated 143B sEVs (Fractions F1‐4), a high protein/low particle count negative control (Fractions F5‐10), proteins that had passed through a 100 kDa cut‐off ultrafiltration device (flow‐through), and a 143B whole cell lysate control were compared for CD81, CD9, and GM130 expression via Western blot. (B) Expression of CD63 and (C) CD81 surface markers was determined using single‐EV flow cytometry for 143B sEVs (pink histogram) and compared to an isotype control for each antibody (blue histogram) or a buffer/PBS plus antibody control to account for antibody aggregates (grey). (D) 143B sEV morphology was examined using TEM and a representative image is shown. (E) Single‐EV flow cytometry was repeated to assess CD63 and (F) CD81 expression in murine K7M2 sEVs as compared to an isotype or buffer/antibody control. (G) The morphology of K7M2 sEVs was also examined using TEM. (H) Flow cytometry analyses are quantified as the percent of sEVs positive for each marker for particles between ∼113 and ∼450 nm based on polystyrene sizing beads.

To assess the purity of our isolated sEV preparations, we comprehensively characterised the expression of canonical sEV markers. We compared our sEV isolate derived from 143B human osteosarcoma cells (fractions 1 through 4 from the qEV2) or an equivalent volume of our low particle count, high protein fractions from the same preparation (5 through 10) for expression of the sEV surface markers CD81 and CD9 and the cellular contamination marker GM130 via Western blot analysis. The 143B sEV isolate (F1‐4) showed a robust presence of both the canonical surface markers CD81 and CD9, whereas fractions 5 through 10 showed minimal expression. Moreover, our sEV isolate (F1‐4) shows no expression of GM130. We compared our sEVs (F1‐4) and fractions 5–10 to two additional controls: proteins that had passed through the membrane of the ultrafiltration device used to isolate our sEVs (proteins <100 kDa) as well as a 143B whole cell lysate control to show reactivity of our negative control antibody GM130 (Figure 2A). Further characterisation of internal sEV proteins and other co‐isolated proteins was performed via an ExoCheck immunoblot (Figure S1B).

Next, we determined the amount of sEVs expressing specific surface markers via single EV flow cytometry. We evaluated both CD63 (Figure 2B) and CD81 (Figure 2C) expression by 143B sEVs and observed nearly 80% positivity. We gated on sEVs based on flow cytometry sizing beads (Figure S2A) and evaluated surface marker expression using both ultracentrifugation (Figure S2B) as well as ExoQuick (Systems Biosciences) precipitation approaches (Figure S2C). We observed a dramatic alteration in the forward and side scatter characteristics of sEVs following exposure to ExoQuick, so we opted to use ultracentrifugation for all wash steps when labelling sEVs (Figure S2C). 143B sEVs were also examined via transmission electron microscopy, which demonstrated the expected size and morphology of sEVs, including a region of central depression as well as evidence of a lipid bilayer (Figure 2D) (Mahgoub and Abdella 2023). These experiments were repeated for murine K7M2 osteosarcoma cell‐derived sEVs, which show similarly high expression of canonical sEV surface markers via flow cytometry (Figure 2E,F) and characteristic sEV morphology via TEM (Figure 2G). Flow cytometry data is quantified in Figure 2H. These data confirm our ability to isolate and concentrate a pure population of sEVs for use in subsequent downstream assays for the validation of different sEV labelling techniques.

### 3.3 PKH26 Labelling Approaches That Introduce Protein Can Form False‐Positive Particles Detectable by Single‐EV Flow Cytometry

Next, we evaluated four different protocols for sEV labelling with PKH26 to determine whether they promote or reduce non‐specific PKH26 particle formation, either self‐aggregation into fluorescent micelles or aggregation with proteins. To do this, we stained 1E10 sEVs with a final PKH26 concentration of 1 µM for 5 min at room temperature in diluent C. A control sample (PBS eluted from the SEC column) was stained in an identical manner. We then transferred stained sEVs or dye vehicle controls into one of four quenching buffers: PBS, 5% dextrose in water (D5W), 10% BSA in PBS or 20% EV depleted FBS in DMEM. Samples were subsequently ultracentrifuged for 70 min at 100,000 × *g* and resuspended in PBS for downstream use.

**FIGURE 3:**
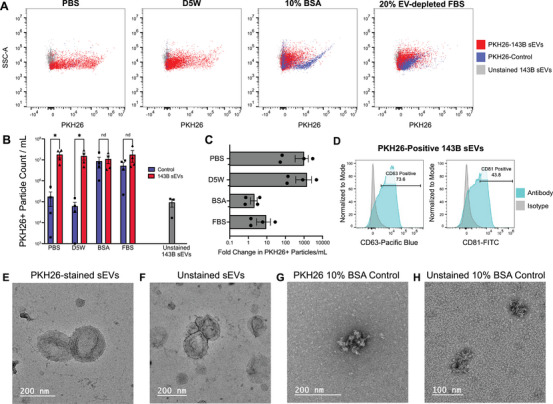
Single sEV flow cytometry shows high levels of false positive dye particle formation in control samples following quenching with either 10% BSA or 20% EV‐depleted FBS. (A) Representative flow cytometry plots are shown for each quenching buffer: PBS, D5W, 10% BSA in PBS, and 20% EV‐depleted FBS in DMEM. Minimal dye particles in control samples are detected when a PBS or D5W quenching buffer is used whereas high levels of dye particles are detected in control samples when 10% BSA or 20% EV‐depleted FBS is used to quench PKH26 staining. Dye control samples are shown in blue, stained sEVs are shown in red, and unstained sEVs are shown in grey. (B) Signals are quantified across four independent staining experiments from 10 µL of sample diluted 1:10 in PBS. Data is shown as counts per millilitre ([raw counts]*10/10 µL) and is compared to a control of unstained sEVs used to set gates (<0.1% PKH26 positivity). Stained sEVs are shown in red, whereas dye controls are shown in blue. Data was tested for normality using a Shapiro‐Wilk test (*α* = 0.05) and upon passing was analysed for significance using a ratio paired *t*‐test and corrected for multiple comparisons using the two‐stage step‐up method of Benjamini, Krieger and Yekutieli with a false discovery rate of <5% (Benjamini et al. 2006). *p* = 0.009783 for PBS quenched samples, and *p* = 0.002359 for D5W quenched samples. (C) The fold difference of PKH26‐labelled sEVs as compared to their respective PKH26 control sample is shown for each quenching buffer, controls are paired for each buffer and experiment day. (D) Single‐EV flow cytometry was performed to determine the percent of PKH26‐stained sEVs that are also positive for CD63 and CD81 as compared to an isotype control for each antibody. Antibody‐stained samples are shown in teal and isotype controls are shown in grey for PBS quenched 143B sEVs stained with PKH26. (E) PKH26‐stained sEVs (quenched with PBS) were examined for morphology via TEM. (F) Unstained sEVs from the same stock used in E also examined via TEM. (G) A representative TEM image of a dye particle from a PKH26 dye‐only control sample quenched with 10% BSA in PBS is shown. (H) TEM image of a control sample with no PKH26 added, but quenched in the same manner as G with 10% BSA in PBS shows small BSA aggregates.

Following this streamlined protocol for sEV labelling, we evaluated the four different quenching buffers on their ability to induce non‐specific PKH26 particles, as previously reported in the literature (Simonsen 2019; Melling et al. 2022; Pužar Dominkuš 2018). We observed efficient sEV labelling with PKH26 across all conditions but substantial non‐specific dye particle formation in control samples when quenched with protein‐containing buffers (Figure 3A‐C). Both 10% BSA in PBS and 20% EV‐depleted FBS in DMEM increased the formation of non‐specific dye particles in control samples to the point that controls were indistinguishable from PKH26 stained sEVs by single‐EV flow cytometry (Figure 3A,B). In contrast, quenching excess dye with protein‐free buffers (PBS or D5W) resulted in an over 1000‐fold difference between the number of PKH26 stained EVs and PKH26 dye particles (Figure 3C). The average signal‐to‐noise ratio of PKH26‐stained sEVs to PKH26 controls when quenched with protein‐containing buffers was less than 10, with ratios as low as 1 (Figure 3C). In other words, quenching with protein‐free buffers decreased non‐specific PKH26 dye particles by nearly 99% as compared to protein‐containing buffers. These results show that the inclusion of protein during sEV staining can result in substantial non‐specific particle formation. We also validated these findings in murine‐derived sEVs and show the phenomenon holds true across multiple sEV sources from different species (Figure S3).

We additionally evaluated the expression of the canonical sEV surface markers CD63 and CD81 on PKH26‐labelled sEV samples to ensure adequate recovery of true sEVs following the labelling process. We maintain relatively high levels of surface marker expression with 73.6% of labelled sEVs quenched with PBS expressing CD63 and 43.8% expressing CD81 (Figure 3D). Although our recovery of true sEVs was lower than our initial input (between 11% and 21% fewer CD63/CD81 expressing particles), this is a substantially higher yield of true PKH26‐positive sEVs following the staining procedure than previously published reports (Melling et al. 2022).

We hypothesised that rather than promoting PKH26 lipid micelle self‐aggregation, protein‐containing buffers may be forming PKH26‐protein aggregates. The size of stained sEVs and PKH26 non‐specific particles were indistinguishable by flow cytometry, so using TEM, we investigated the morphology of stained sEVs, unstained sEVs, dye particles, and normal protein aggregates (Figure 3E‐H). PKH26 staining of EVs using PBS as a quenching buffer resulted in similar morphology to unstained sEVs (Figure 3E,F). Qualitatively, PKH26‐labelled sEVs looked slightly paler, more flattened, with a less distinct outer membrane. However, further investigations with cryogenic electron microscopy would be required to maintain sEV morphology for direct comparisons. Both stained and unstained sEVs appear morphologically distinct from the dye particles shown in Figure 3G that formed during quenching with 10% BSA in PBS. To compare to normal BSA aggregates in the absence of PKH26, we prepared a sample in an identical manner as our normal dye control quenched with 10% BSA in PBS, however, no PKH26 was added to the sample (Figure 3H). The particles observed in Figure 3G,H were morphologically similar to one another but highly distinct from sEVs. No sEV or micelle‐like structures were observed in the 10% BSA quenched PKH26 dye control sample, supporting the hypothesis that the particles formed are aggregates of PKH26 and protein rather than self‐aggregation of lipid micelles. The aggregates observed in the dye control sample were similar to unstained BSA aggregates, but qualitatively, they appeared larger, more numerous, and there was less of a protein‐rich background on the carbon‐coated grid, suggesting more incorporation of BSA with PKH26 into larger aggregates. However, further morphological investigation would be necessary to distinguish these particles.

Current manufacturer protocols recommend the inclusion of 10% BSA as a ‘quenching’ solution in the PKH26 staining process and other reports have suggested quenching the dye signal with EV‐depleted FBS (Melling et al. 2022; Chen et al. 2023; Pužar Dominkuš 2018). Though the inclusion of this step can adsorb unbound PKH26 dye, our findings show that it can promote sEV‐sized protein‐dye aggregates that are not able to be separated from sEVs via ultracentrifugation. We additionally investigated whether column‐based techniques marketed to remove non‐specific dye particles from true, labelled EVs could eliminate these particles but found that they were equally inefficient at particle removal in control samples as ultracentrifugation (Figure S4A). The use of PBS or D5W as a quenching buffer can prevent the formation of these aggregates in the first place, avoiding the need for additional techniques to separate the labelled sEVs from the aggregates after labelling.

In order to further validate that we are generating true, labelled sEVs using a protein‐free quenching approach and not dye‐protein aggregates generated from residual free protein in the sEV isolate, we labelled additional control samples in which exogenous protein was added to PBS from the SEC column at an equivalent concentration as is present in an sEV sample (50.7 µg/mL). The level of PKH26 positive particles in our protein‐matched samples did not significantly differ from PBS controls (Figure S4B,C). This finding supports the conclusion that our sEV isolates are highly concentrated and pure, and the level of free protein within the isolates is not contributing to the high quantity of PKH26 positive sEVs observed after labelling. Moreover, we observed no difference in the diameter of our sEVs following staining as measured by both nanoparticle tracking analysis (Figure S4D) or forward and side scatter characteristics in single‐EV flow cytometry (Figure S4E,F).

### 3.4 Non‐Specific PKH26 Dye Particles Are Taken Up by Cells In Vitro and Are Indistinguishable From PKH26‐Labelled sEVs

**FIGURE 4:**
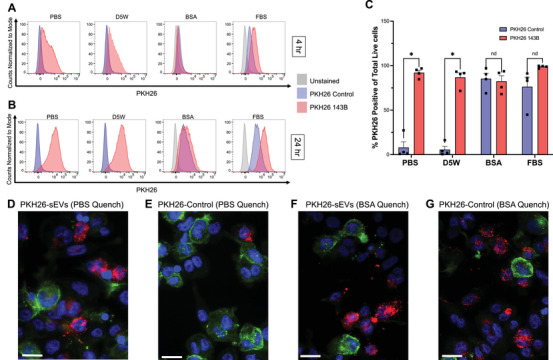
False positive PKH26 particles can be internalised and detected in THP‐1 monocytes. THP‐1 cells were treated with 1E9/mL 143B sEVs labelled with PKH26 or a matched PKH26 control from each dye quenching condition: PBS, D5W, 10% BSA in PBS, or 20% EV‐depleted FBS in DMEM. (A) Representative flow cytometry histograms of PKH26 fluorescence intensity in THP‐1 cells after 4 h of treatment on the x‐axis compared to normalised counts on the y‐axis. Single, live THP‐1 cells were gated on based on a live/dead stain prior to examining PKH26 fluorescence intensity. Unstained cells are plotted in grey, cells treated with PKH26 controls are in lavender, and cells treated with PKH26‐stained 143B sEVs are in pink. (B) Representative histograms of THP‐1 uptake of sEVs or dye particles after 24 h. (C) The percent of THP‐1 cells positive for PKH26 for each group at 24 h is quantified; cells treated with dye controls are shown in lavender and labelled sEVs shown in pink. Differences between control and sEV samples were analysed with a Shapiro‐Wilk test for normality, which samples passed, followed by an unpaired *t*‐test with a Welch's correction and correction for multiple comparisons using the two‐stage step‐up method of Benjamini, Krieger and Yekutieli with a false discovery rate of 5%; *p* = 0.000265 for PBS quenched samples, *p* = 0.000027 for D5W quenched samples (*n* = 4 independent experiments per group) (Benjamini et al. 2006). Fluorescent imaging was performed on THP‐1 cells grown on coverslips and treated with sEVs or dye controls for 24 h. Nuclei were stained with DAPI (blue), F‐actin with ActinGreen 488 (green), and sEVs or dye particles with PKH26 (pink). Representative fluorescence images of THP‐1 cells treated with PKH26‐labelled sEVs quenched with PBS (D), a PKH26‐labelled dye control quenched with PBS (E), PKH26‐labelled sEVs quenched with 10% BSA in PBS (F) or a PKH26‐labelled dye control quenched with 10% BSA in PBS (G). White bars indicate 25 µm.

To determine if the PKH26 dye particles generated by quenching with protein‐containing buffers impacted in vitro cell culture experimental results, we evaluated sEV and dye particle uptake by THP‐1 monocytes. We incubated cells for either 4 or 24 h with PKH26‐labelled osteosarcoma 143B sEVs or a matched PKH26 control to evaluate cellular uptake (Figure 4). We observed dye particle and sEV uptake by THP‐1 cells after 4 h (Figure 4A) by flow cytometry and further uptake by 24 h (Figure 4B). We compared all four quenching conditions and observed that control samples quenched with either PBS or D5W resulted in low background signal, which was nearly indistinguishable from the unstained controls at both 4 (Figure 4A) and 24 h (Figure 4B). On the other hand, treating THP‐1 cells with PKH26 control samples quenched with 10% BSA in PBS or 20% EV‐depleted FBS in DMEM resulted in high PKH26 positivity of cells, making it nearly impossible to distinguish cells that had taken up PKH26‐labelled sEVs versus their matched PKH26 dye control. Upon quantification of the percent of THP‐1 cells positive for PKH26, there was a significant increase in PKH26 positivity (∼82%) between sEV‐treated and control samples for protein‐free quenching buffers. However, there was no significant difference in the percent of PKH26‐positive cells between sEV‐treated and controls for protein‐containing buffers. Quenching the PKH26 staining with 10% BSA in PBS resulted in the worst signal‐to‐noise ratio between sEV‐treated and control samples, with controls having 3% more PKH26 positive cells than sEV‐treated samples (Figure 4C).

Additionally, we also used fluorescence microscopy to examine THP‐1 cell uptake of PBS quenched sEVs and 10% BSA in PBS quenched sEVs after 24 h (Figure 4D‐G). We observed a strong positive PKH26 signal in sEV treated cells quenched with PBS (Figure 4D) as compared to a matched PBS quenched control (Figure 4E). Although we observed PKH26 signal within cells treated with BSA‐quenched sEVs (Figure 4F), we also observed a strong PKH26 signal in our BSA‐quenched control samples as well (Figure 4G), similar to flow cytometric results. Full‐sized images of each THP‐1 treatment group are included in Figure S5. These data demonstrate that quenching PKH26 with a protein‐containing buffer, like 10% BSA in PBS, results in the formation of non‐specific dye particles that are taken up by cells similarly to sEVs, significantly confounding in vitro studies investigating sEV cell uptake.

### 3.5 PKH26‐Labelled sEVs and Dye Particles Are Detectable Within Lymph Nodes in Mice

In order to determine how these different PKH26 labelling approaches perform in vivo, we evaluated sEV biodistribution in mouse popliteal lymph nodes. To do this, we injected 2.5E10 PKH26‐labelled K7M2 osteosarcoma sEVs and a matched PKH26 control for each of the four dye quenching approaches into the footpads of CD‐1 mice. We then investigated PKH26 positivity within cells of the draining, popliteal lymph node 24 h post injection via intravital (IVIS) imaging and spectral flow cytometry. We also developed a 10‐colour antibody panel to distinguish cells of the murine lymph node to determine the specific cellular localisation of the PKH26 signal.

**FIGURE 5:**
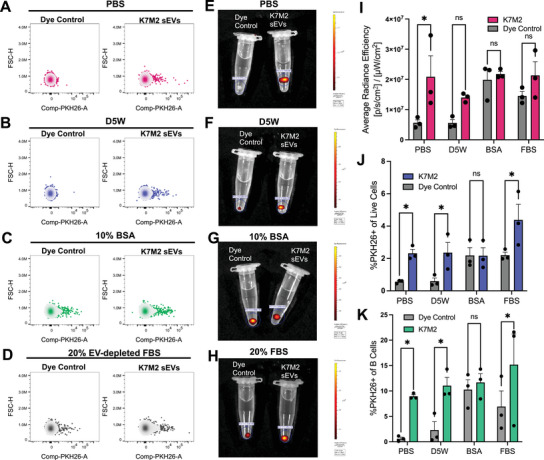
Detection of PKH26 positive cells within murine lymph nodes following labelled sEV injection. Mice were given footpad injections of 2.5E10 PKH26‐labelled K7M2 sEVs or a PKH26 control from each of the four quenching buffer protocols and popliteal lymph nodes were harvested 24 h later. (A) Mice were treated with either PKH26‐sEVs quenched with PBS (right) or a matched dye control (left) and lymph nodes were processed for flow cytometry. Lymph node cells were first gated for live, single cells and then PKH26 fluorescence intensity per cell is shown on the x‐axis as compared to forward scatter height (size) on the y‐axis. The remaining three quenching techniques were assessed as in A and PKH26 positive lymph node cells are shown for sEV and control treatments quenched with D5W (B), 10% BSA in PBS (C), and 20% EV‐depleted FBS in DMEM (D). Whole excised lymph nodes were imaged prior to the preparation of single cell suspensions for PKH26 fluorescence using the IVIS imaging system and a representative image for each treatment group is shown in (E–H). (I) Average radiance efficiency for each lymph node was quantified across quenching methods (*n* = 3 lymph nodes/group). Data were analysed using multiple unpaired *t*‐tests; *p* = 0.004717. (J) The percent of live lymph node cells positive for PKH26 assessed by flow cytometry is quantified (*n* = 3/group). Data were analysed using multiple unpaired *t*‐tests; *p* = 0.022390 for PBS quenched samples, *p* = 0.022174 for D5W quenched samples and *p* = 0.006305 for EV‐depleted FBS quenched samples. (K) Live lymph node cells were gated for CD45 positivity and then B220 positivity to quantify the percent of B cells positive for PKH26 (*n* = 3/group). Data was analysed using multiple unpaired *t*‐tests; *p* = 0.043415 for PBS quenched samples, *p* = 0.035204 for D5W quenched samples and *p *= 0.045555 for EV‐depleted FBS quenched samples.

A strong PKH26 signal was detected in the lymph node at 24 h by flow cytometry (Figure 5A‐D) as well as by ex vivo IVIS imaging (Figure 5E‐H). The level of non‐specific dye particle formation followed the same patterns as seen in cell‐based assays, with high levels of background signal in control samples that had been quenched with protein‐containing buffers (Figure 5C,D,G,H). Footpads that had received PKH26 labelled sEVs had at least 2% of lymph node cells positive for PKH26 fluorescence across conditions (Figure 5J). On the other hand, mice that had received dye controls quenched with protein‐free buffers (PBS or D5W) had minimal background signal, whereas quenching with protein‐containing buffers resulted in PKH26‐positive cells at levels similar to sEV treated lymph nodes (Figure 5I,J).

Individual cells of the lymph node were distinguished based on a flow cytometry panel to determine the cell specificity of sEVs (Figure S6). Of the various cell types found in the lymph node, the majority of PKH26 positive cells were CD45 positive and B220 positive, suggesting that sEVs are specifically internalised by B cells by 24 h (Figure S5D). The largest fold increase in B‐cell specific uptake was observed in sEVs quenched with either PBS or D5W compared to their respective PKH26 dye controls (Figure 5K). These data suggest that both labelled sEVs and non‐specific PKH26 dye particles persist in vivo and associate with specific cell types in the murine lymph node. Our findings further support the notion that the elimination of PKH26 dye particles is critical to ensuring appropriate evaluation of sEV biodistribution in biological systems as they are indistinguishable from sEVs by traditional flow cytometry approaches.

## 4 Discussion

Despite the rapid expansion of EV research, techniques for labelling EVs remain unstandardised and controversial in the literature. The 2023 Minimal Information for Studies of Extracellular Vesicles (MISEV2023) report provides an outline of suggested steps required to validate a labelling approach and recognises the drawbacks of different techniques (Welsh et al. 2024). However, a specific, gold standard approach is not provided as the wide variety of EV applications may necessitate different methodologies. The steps required to properly validate a given approach are highly comprehensive and often not fully reported in published reports on in vitro or in vivo uptake of labelled EVs. Thus, it is critical that further validation of labelling approaches be performed and thoroughly reported to minimise the variation and lack of standardisation across the field.

We aimed to evaluate the limitations of the widely used lipophilic membrane dye PKH26 and define a streamlined approach to effectively use PKH26 to label sEVs for in vitro and in vivo applications. Our findings contribute to an emerging body of evidence that suggests subtle changes in experimental conditions, which often go unreported, can vastly alter experimental outcomes and conclusions in EV studies. Our results show that the inclusion of protein‐containing buffers during EV labelling with PKH26 leads to the production of non‐EV associated dye particles that can contribute to false positive results. Moreover, we show that by staining a highly concentrated, pure population of sEVs and eliminating protein from buffers during the staining process, we can largely overcome this major limitation of PKH26. Previous studies have noted the propensity for LMDs, like PKH26, to produce non‐specific, fluorescent particles that are indistinguishable from EVs. We have built on this foundational knowledge by determining conditions that lead to undesirable contaminants, optimised a staining protocol that limits particle formation, and benchmarked its performance in cell‐based assays as well as in a mouse model (Melling et al. 2022; Pužar Dominkuš 2018).

To do this, we evaluated four different sEV labelling approaches to determine conditions that either promote or reduce non‐specific PKH26 dye particle formation. We first thoroughly validated our sEV isolation process (Figure 1) and ensured a high purity and concentration of isolated sEVs that expressed canonical surface and cytosolic markers demonstrated through single‐EV flow cytometry, western blot and antibody array immunoblot (Figures 2 and S1). After optimising isolation of a pure population of sEVs, we next evaluated four different approaches for removing unbound PKH26 dye following labelling of sEVs or sEV‐free controls. Our results show that including protein in the PKH26 removal or ‘quenching’ step, either 10% BSA in PBS or 20% EV‐depleted FBS in DMEM, can promote the formation of PKH26 dye aggregates that are largely indistinguishable from sEVs (Figure 3). The morphology of these dye aggregates could only be distinguished through TEM, in which we observed jagged, asymmetrical aggregates that appeared highly distinct from the round, membrane‐bound PKH26‐labelled sEVs (Figure 3E‐H). In contrast, quenching labelled sEVs or controls with a protein‐free buffer, such as PBS or D5W, resulted in minimal dye aggregates. Single‐EV flow cytometry showed a nearly 1000‐fold increase in the number of labelled sEVs as compared to dye particles using a protein‐free protocol, whereas the inclusion of protein resulted in equivalent numbers of non‐specific dye‐protein particles as labelled sEVs (Figure 3B,C). These results emphasise that a simple change in buffer condition to include protein, which is included in both manufacturer and previously published protocols, can result in the production of non‐specific PKH26 aggregates equal in concentration and relative size to labelled sEVs (Pužar Dominkuš 2018).

It is critically important to understand the implications that non‐specific dye particles can have in model systems, rather than just at the single sEV level. Thus, we evaluated the ability of sEVs and dye particles to be taken up and visualised in cells (Figure 4). THP‐1 monocytes indiscriminately took up labelled sEVs as well as non‐specific PKH26 particles. Samples that had been quenched with protein‐containing buffers could not be distinguished from their respective control samples at 24 h by flow cytometry (Figure 4C). Fluorescent imaging shows internalisation of both labelled sEVs and non‐specific BSA‐PKH26 dye particles by monocyte‐derived macrophages (Figure 4D‐G). These data emphasise the importance of including proper controls when labelling sEVs as cells are capable of taking up non‐specific dye particles with a similar efficiency to true sEVs.

To determine if these in vitro findings of false‐positive dye particle uptake translated to an in vivo setting, we evaluated each of the sEV labelling approaches in a mouse model. We successfully identified PKH26 positive cells in murine lymph nodes 24 h following a foot pad injection of labelled sEVs. Our in vivo results mimic our in vitro findings in cell culture, showing that removing protein from the staining process results in a significantly higher signal‐to‐noise ratio of labelled sEVs to their matched dye control (Figure 5). Labelled sEVs and non‐specific dye particles were both primarily internalised by B cells of the lymph node, and we observed no difference in the level of PKH26 signal in B cells between sEVs and controls quenched with protein‐containing buffers (Figures 5 and S6). We propose this model as an effective way to evaluate a staining approach for use in an in vivo system before using sEVs to answer true biological questions regarding trafficking and cellular uptake.

In summary, our findings identify a propensity for PKH26 to interact with protein and form non‐specific fluorescent particles that are indistinguishable from labelled EVs. With the elimination of protein during any washing steps, the formation of these protein‐dye aggregates can be avoided while maintaining efficient labelling of true sEV membranes. The approach for PKH26 labelling described here is likely dependent on beginning the staining process with a highly concentrated, pure and fresh sample of EVs. We predict, based on our findings, that limiting contaminating, non‐EV associated protein in the initial EV isolate is essential to reduce protein‐dye aggregates and label true EVs. Moreover, we recommend that the concentration of PKH26 is optimised for a given starting concentration of EVs to reduce excess dye during staining. Finally, it may be necessary to improve upon the negative controls used for a given sEV preparation if that preparation contains an excess of non‐EV protein (>50–60 µg). A better control to use in this scenario would be a sample with a matched protein concentration to the sEVs of interest like those evaluated in Figure S4B,C. Ultimately, we feel that with proper controls and precautions, PKH26, and likely other LMDs, can used effectively for studies of cellular uptake and biodistribution of sEVs.

Several other considerations should be noted when using LMDs, like PKH26, for investigations of sEVs in vitro and in vivo. First, a limitation that has been described of LMDs is that dye intercalation into the membrane of sEVs could affect their size and ability to traffic to their true biological destination in vivo (Welsh et al. 2024; Dehghani et al. 2020). Though we observed no differences in sEV size or morphology post‐staining via nanoparticle tracking analysis, flow cytometry or qualitatively by TEM, we cannot exclude the possibility that LMDs could alter in vivo trafficking capabilities based on subtle size or membrane alterations (Figures 3 and S4D‐F). Another concern surrounding the use of LMDs, particularly in vivo, is that their stability within an sEV membrane has not been well described. It has been suggested that LMDs may be able to desorb from the membrane and then associate with other membranes or hydrophobic bindings pockets of plasma proteins, like albumin (Simonsen 2019; Curry et al. 1998). Though manufacturers claim stable incorporation of PKH26 into membranes, the true level of desorption from sEVs is unknown and poses concerns for in vivo experiments. Our in vivo biodistribution study shows the specificity of sEVs and dye particles for B cells rather than a diffuse staining of all cells of the lymph node (Figure 5K). These findings support the notion that PKH26 is likely not rapidly dissociating from the membrane and indiscriminately labelling cells. These results also provide some of the first evidence that dye‐protein complexes are stable in an in vivo system and can be detected within cells just like sEVs, confounding experimental results. Our in vitro experiments investigating THP‐1 uptake were performed under serum‐free conditions, so we were unable to evaluate the level of desorption and association with serum proteins, which is a limitation of these studies. However, we did not observe diffuse staining of cells in vitro, but rather punctate signals, again supporting the idea that labelled sEVs remain intact and PKH26 is not desorbing from the membrane and indiscriminately labelling cell membranes.

There are several additional investigations that would expand upon the findings of this presented study in the future. First, in order to truly evaluate the level of LMD desorption and reassociation with proteins, it would be necessary to generate a transgenic cell line with a labelled canonical sEV surface marker to distinguish labelled EVs from dye particles. In our hands, cell lines could be successfully transduced with an sEV reporter transgene CD63‐RFP, but the purified sEVs isolated from fractions 1 through 4 from the qEV2 SEC column, while expressing CD63, were not positive for RFP. Thus, it was out of the scope of these studies to pursue further analysis of desorption. Additionally, to enhance the translatability of our findings to other systems, we propose investigating the ability of PKH26 to label EVs of different subtypes and from different sources or isolation approaches, such as differential ultracentrifugation or bead‐based purification, to determine in which instances LMDs may perform better or worse. Finally, investigations into sEV stability over time prior to and after labelling with LMDs would be pertinent to long‐term studies.

In conclusion, our results demonstrate how different approaches to LMD‐based sEV labelling can result in highly divergent experimental outcomes, including false positive results that would inform erroneous conclusions. Protein‐containing buffers increase the formation of PKH26 dye aggregates to the point that control samples are indistinguishable from sEV samples. Moreover, we show that these particles behave similarly to sEVs in vitro and in vivo, resulting in false positive signals if appropriate dye controls are not used. Though PKH26 and other LMDs carry many limitations, the current state of sEV research has not identified a gold standard, and these LMDs continue to be widely used (Costa‐Silva et al. 2015; Ruan et al. 2024; Guo et al. 2022; Wei et al. 2020; Herman et al. 2023). Transgenic cell lines or even rodent models with fluorescent protein‐tagged EV surface markers overcome many of these challenges by producing pre‐labelled EVs (Yoshimura et al. 2016; Garcia et al. 2015). However, these systems are expensive to generate and not possible in studies using primary cells or sEVs derived from clinical samples. Thus, it is important to thoroughly evaluate multiple approaches to sEV labelling for different applications. Our work provides a comprehensive assessment of PKH26 sEV labelling methods and defines an approach to reduce dye particle formation for use in downstream experiments.

## Author Contributions

**Laurel Haines**: conceptualization (supporting), data curation (lead), formal analysis (lead), investigation (lead), methodology (lead), project administration (equal), software (equal), supervision (equal), validation (equal), visualization (lead), writing ‐ original draft (lead), writing ‐ review & editing (lead). **Alex Baeckler**: data curation (supporting), formal analysis (supporting), methodology (supporting), visualization (supporting), writing ‐ review & editing (supporting). **Sophi Schofield**: data curation (supporting), formal analysis (supporting), methodology (supporting), visualization (supporting), writing ‐ review & editing (supporting). **Eric Palmer**: conceptualization (supporting), investigation (supporting), methodology (supporting), writing ‐ review & editing (supporting). **Bradley Guilliams**: formal analysis (supporting), methodology (supporting), resources (supporting), software (supporting), writing ‐ review & editing (supporting). **Melinda Meyers**: formal analysis (supporting), methodology‐Supporting), resources (supporting), software (supporting), writing (review & editing (supporting). **Daniel Regan**: conceptualization (lead), data curation (supporting), formal analysis (equal), funding acquisition (lead), investigation (equal), methodology (equal), project administration (lead), resources (lead), software (lead), supervision (Lead), validation (supporting), visualization (supporting), writing ‐ original draft (supporting), writing ‐ review & editing (supporting).

## Conflicts of Interest

The authors declare no conflicts of interest.

## Supporting information



Supporting Information

## Data Availability

The data that support the findings of this study are available from the corresponding author upon reasonable request.
