# Supplementary material for: Non‐Specific Particle Formation During Extracellular Vesicle Labelling With the Lipophilic Membrane Dye PKH26
Source: J Extracell Vesicles. 2025 May 19;14(5):e70079. doi: 10.1002/jev2.70079 (PMC12087298; doi:10.1002/jev2.70079)
Supplement: Supplementary file 1 — Supporting Information [file JEV2-14-e70079-s001.docx]

**Supplemental Figures**


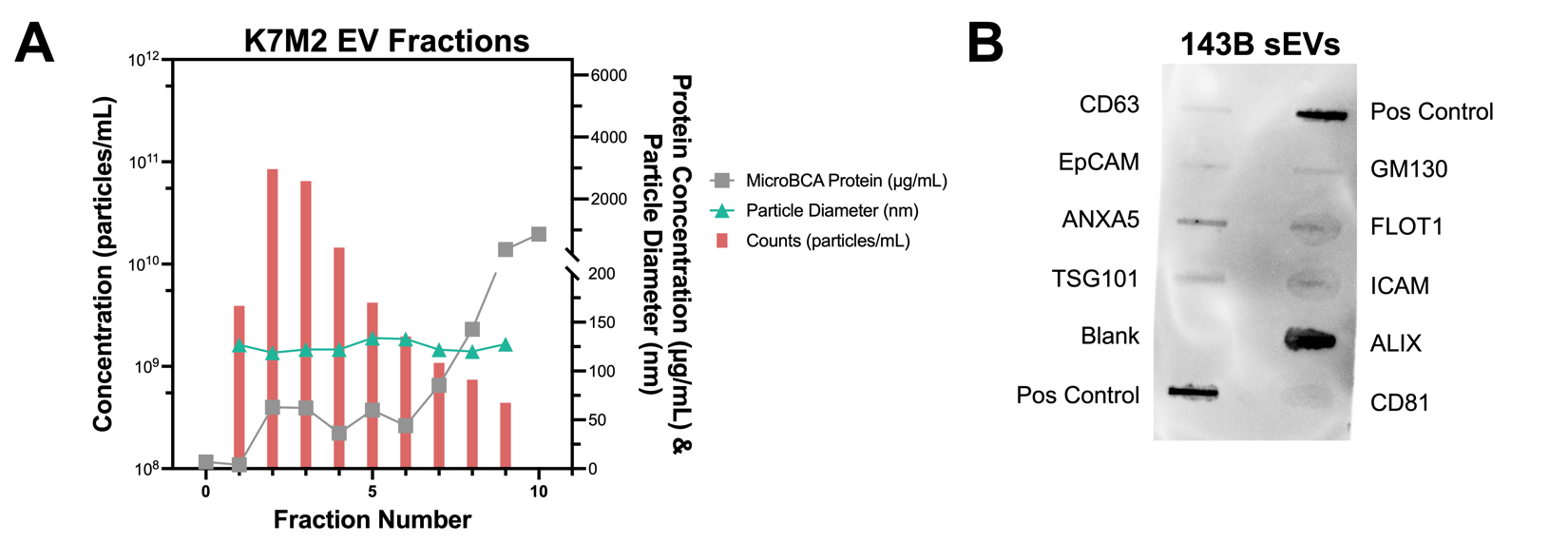


**Supplemental Figure 1.** *Validation of sEV isolation and surface marker expression***. (A)** Particle counts per mL (pink columns), particle diameter in nm (green triangles), and protein concentration (µg/mL) determined via microBCA (gray squares) were assessed across all fractions of murine K7M2 EVs isolated on a qEV2 column. The column void volume is labeled as fraction 0. This data reflects the purity, quantity, and size of isolated EVs across the SEC fractions. **(B)** An ExoCheck immunoblot of 143B sEVs shows positivity for a range of sEV markers known to be within sEVs and on the sEV surface.


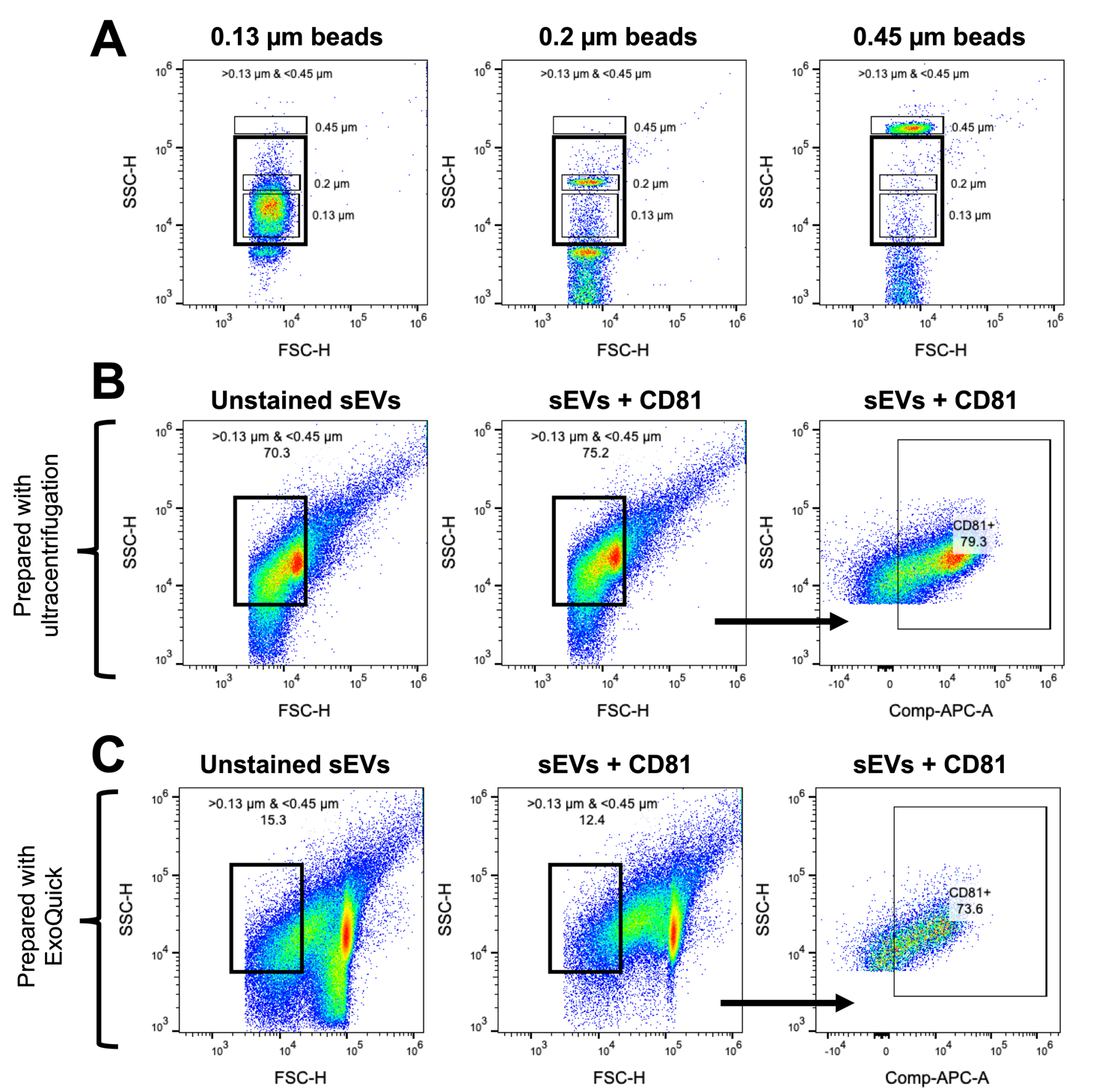


**Supplemental Figure 2**. *Single sEV flow cytometry gating schemes and comparison of ultracentrifugation versus ExoQuick precipitation*. **(A)** 0.13 µm, 0.22 µm, and 0.45 µm polystyrene beads were detectable via forward- and side-scatter on the flow cytometer and used to set size-based gates for sEV samples to isolate particles between 0.13 µm and 0.45 µm in size. **(B)** Forward-scatter versus side-scatter properties of unstained sEVs, sEVs incubated with anti-CD81-APC antibody, and finally APC positivity versus side-scatter of sEV sized particles prepared with ultracentrifugation. **(C)** sEVs are compared for forward and side scatter properties as in B, but prepared using ExoQuick-TC (Systems Biosciences) precipitating agent rather than ultracentrifugation according to manufacturer recommendations.


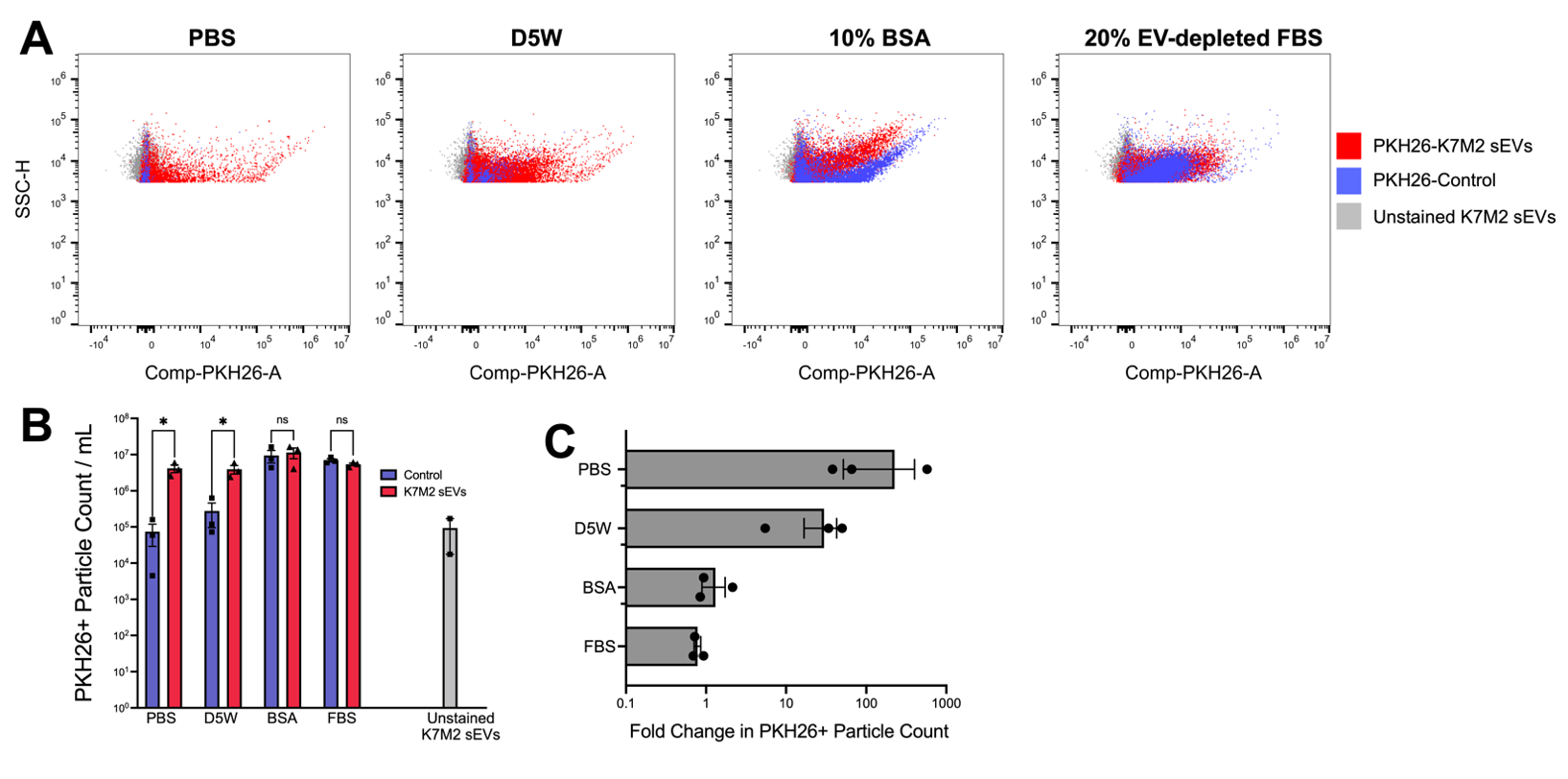


**Supplemental Figure 3.** *Single sEV flow cytometry of K7M2 sEVs shows significant dye particle formation in protein-containing conditions.* **(A)** Representative flow cytometry plots for each method of dye quenching: PBS, D5W, 10% BSA in PBS, and 20% sEV-depleted FBS in DMEM, show dye particle formation in control PKH26 dye samples when quenched with 10% BSA or 20% EV-depleted FBS in DMEM. Dye control samples are in blue, stained K7M2 sEVs are in red, and an unstained K7M2 control is shown in gray**. (B)** Signals are quantified across 3 independent staining experiments from 20 µL of sample diluted 1:10 for quantification, stained K7M2 sEVs columns are red and dye controls are blue. Data is shown as PKH26 positive particles per mL compared to an unstained control and was tested for normality using a Shapiro-Wilk test (α=0.05) and upon passing was analyzed for significance using a ratio paired t-test. **(C)** The fold change of PKH26-labeled K7M2 sEVs per mL as compared to their respective PKH26-only control is shown for each quenching condition.


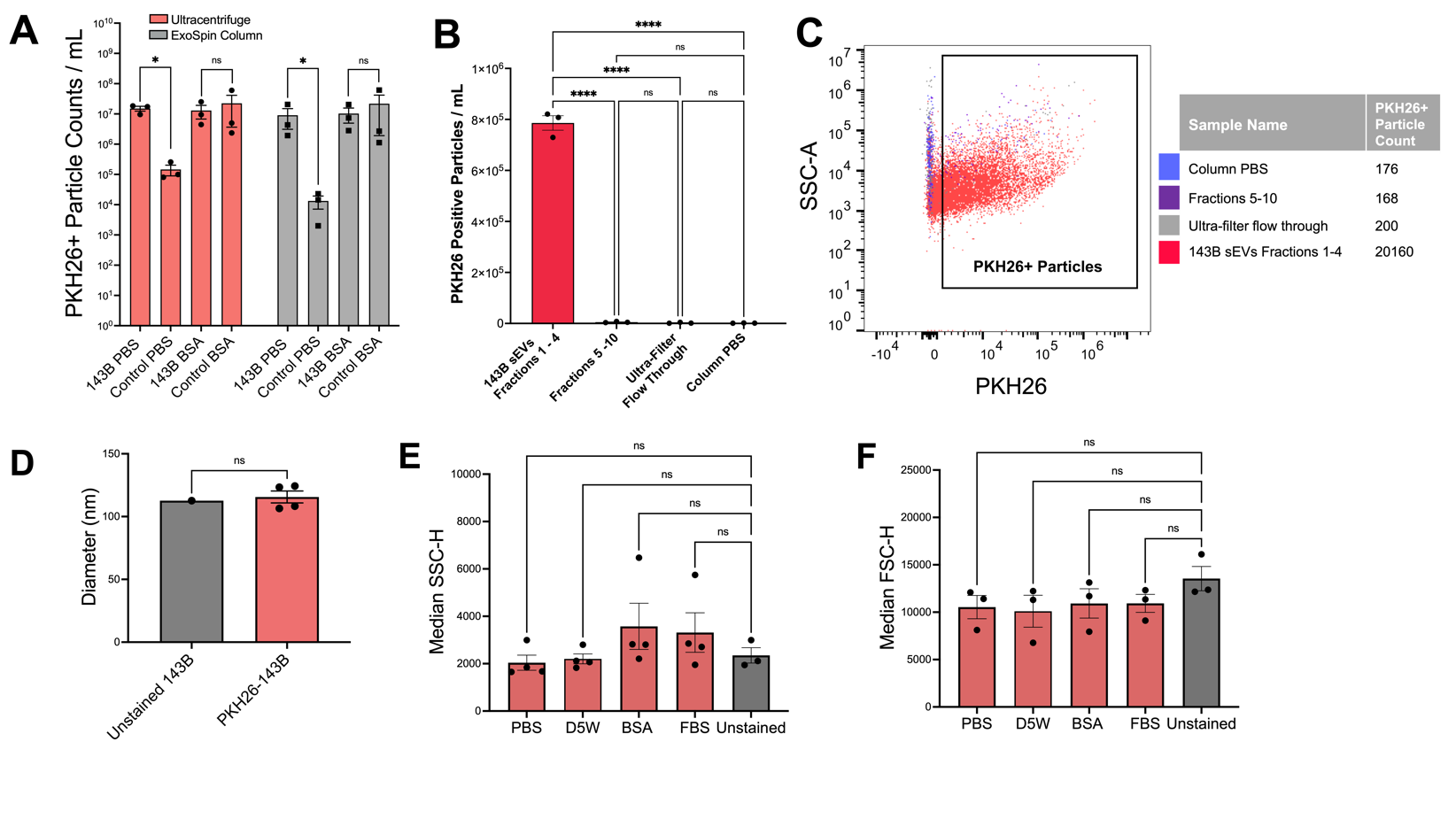


**Supplemental Figure 4.** *Analysis of protein aggregate formation, removal strategies and labeled sEV characteristics.* **(A)** 143B sEVs or matched vehicle controls were stained with PKH26 and quenched with either PBS or 10% BSA in PBS. Removal of non-specific dye particles was attempted with either ultracentrifugation or by using Exosome Spin Columns (MW 3000) Invitrogen^TM^. Both ultracentrifugation and column-based purification failed to remove non-specific dye particles generated in 10% BSA quenched samples. Data is shown as particles per mL as measured by single EV flow cytometry and analyzed using paired t-tests. **(B)** The concentration of protein within a 143B sEV sample was replicated in a sample of column-derived PBS with exogenous protein added from fractions 5 through 10 (high protein, low particle count fractions) or the proteins that passed through the ultra-filter device membrane (ultra-filter flow through, <100 kDa). The samples were stained in an identical manner as 143B sEVs derived from fractions 1 through 4 and assessed for non-specific PKH26 particle formation via single-EV flow cytometry. There was no significant difference between the column-derived PBS control and the protein matched controls in concentration of non-specific particles. **(C)** A representative flow cytometry plot of PKH26 positive particles in 10 µL of sample for labeled 143B sEVs, protein concentration-matched controls, and a column-derived PBS control with particle counts quantified in the table on the right. **(D)** The diameter of freshly isolated 143B sEVs as compared to PKH26-labeled sEVs generated using each of the four labeling approaches measured via nanoparticle tracking analysis with a ParticleMetrix ZetaView. **(E)** Single sEV flow cytometry comparing the side-scatter characteristics of unstained sEVs with PKH26-labeled sEVs using each of the four approaches. **(F)** Single sEV flow cytometry comparing the forward-scatter characteristics of unstained sEVs with PKH26 labeled sEVs.


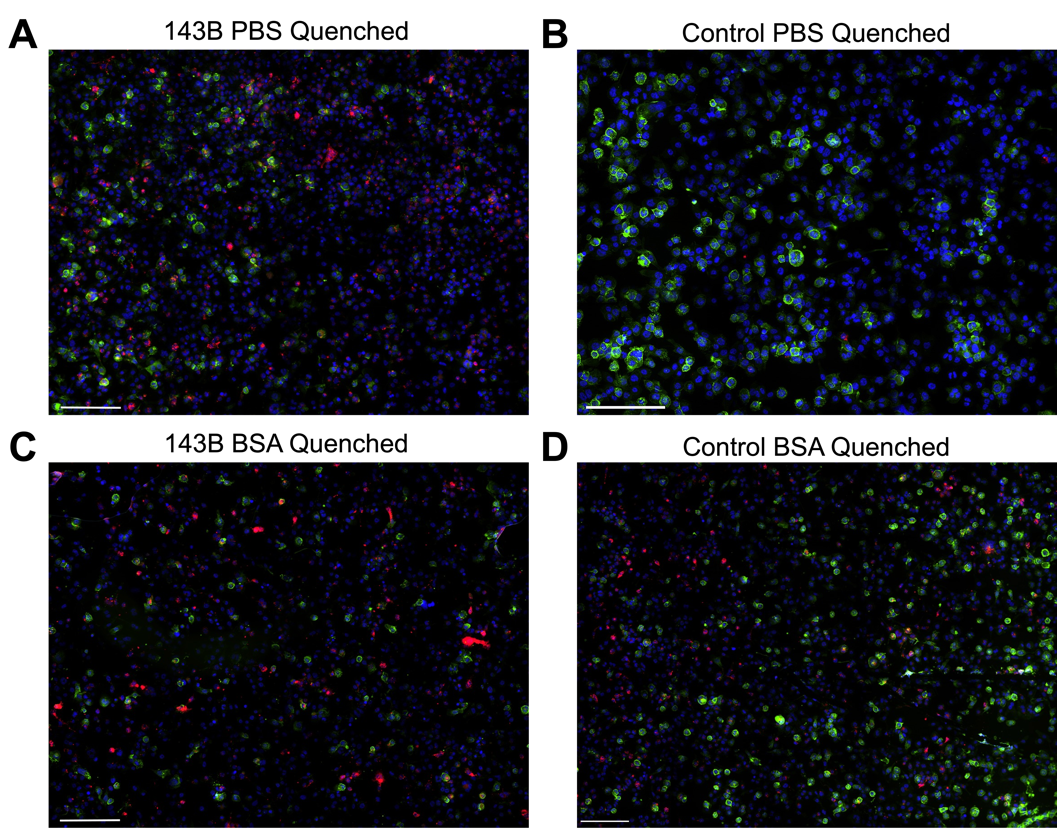


**Supplemental Figure 5.** *Full-sized fluorescence microscopy images of PKH26-sEV uptake by THP-1 macrophages.* THP-1 cells were incubated on coverslips with PKH26-labeled 143B sEVs labeled using either a PBS quenching approach or a 10% BSA in PBS quenching approach. Cells were then stained with ActinGreen 488 for F-actin (Green), DAPI for nuclei (blue), and examined for PKH26 signal (pink) via fluorescence microscopy. **(A)** PKH26-labeled sEVs quenched with PBS. **(B)** Vehicle PKH26 Control quenched with PBS. **(C)** PKH26-labeled sEVs quenched with 10% BSA in PBS. **(D)** Vehicle PKH26 Control quenched with 10% BSA in PBS. Images shown in Figure 4 were derived from these full-sized images. White bars indicate 100 µm.


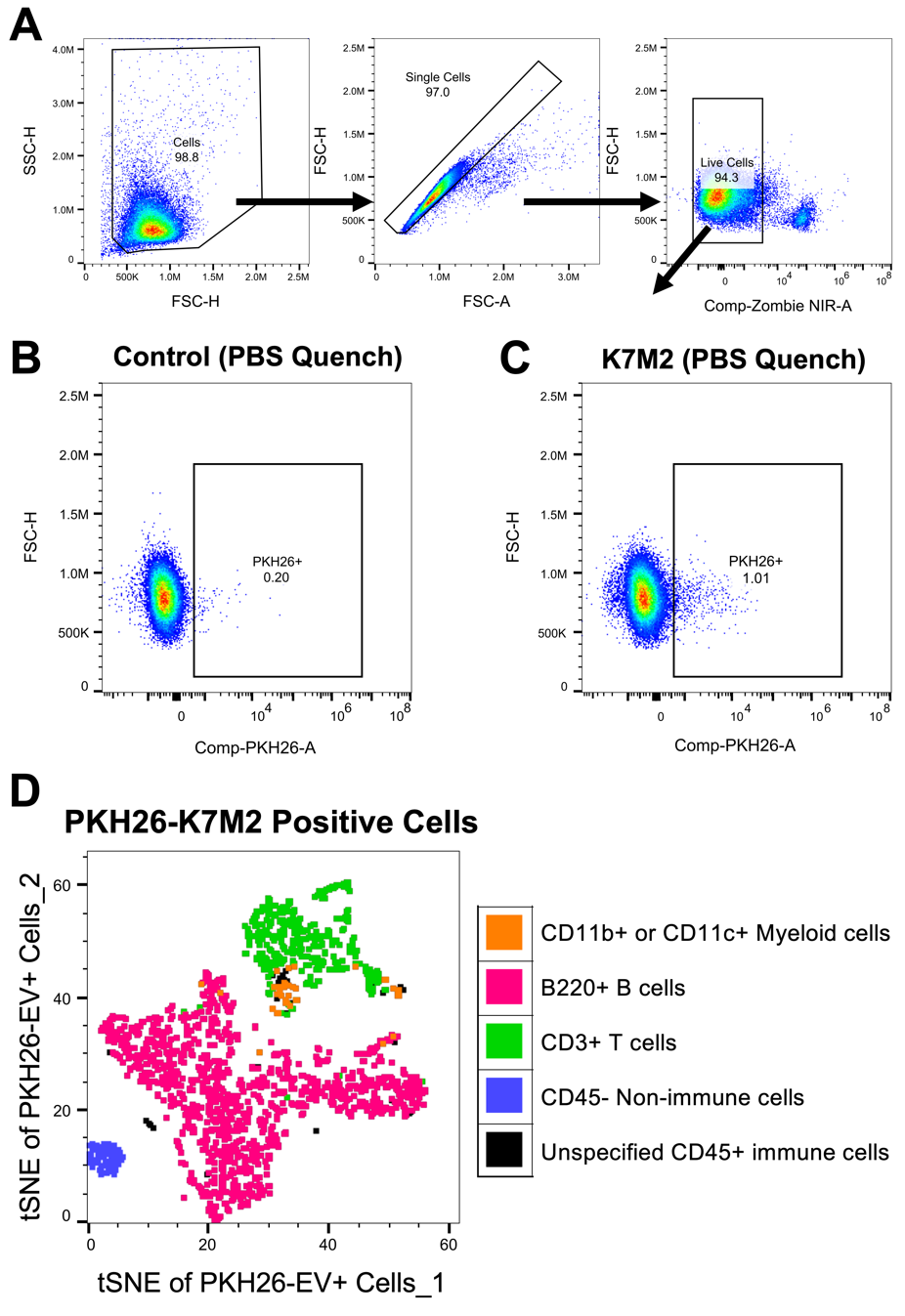


**Supplemental Figure 6.** *Comprehensive evaluation of PKH26-positive cells within mouse lymph nodes and flow cytometry gating scheme.* **(A)** Intact cells were gated from debris via forward and side scatter parameters, then gated for single cells by forward-scatter height versus forward-scatter area, and then gated for live cells (negative for Zombie-NIR live/dead discriminating dye). **(B)** All single, live lymph node cells were plotted for PKH26 fluorescence for mice receiving a foot pad injection of a PKH26 control quenched with PBS as compared to **(C)** an injection of PKH26-stained K7M2 sEVs. **(D)** All PKH26 positive lymph node cells from four separate lymph nodes treated with PKH26-K7M2 sEVs quenched with protein-free buffers were concatenated and analyzed using unsupervised clustering via TSNE to determine cell types positive for PKH26 signal. The majority of cells positive for PKH26 were positive for CD45 and B220 surface markers suggesting PKH26 sEVs may be colocalizing with B cells (pink).
